# The gap in human resources to deliver the guaranteed package of prevention and health promotion services at urban and rural primary care facilities in Mexico

**DOI:** 10.1186/s12960-017-0220-5

**Published:** 2017-08-03

**Authors:** Jacqueline Elizabeth Alcalde-Rabanal, Gustavo Nigenda, Till Bärnighausen, Héctor Eduardo Velasco-Mondragón, Blair Grant Darney

**Affiliations:** 1 Center for Health Systems Research, National Institute of Public Health - Instituto Nacional de Salud Pública, Universidad No. 655 Colonia Santa María Ahuacatitlán, Cerrada Los Pinos y Caminera, CP 62100 Cuernavaca, Morelos Mexico; 2 Partners in Health, Calle Primera Poniente Sur # 25, Angel Albino Corzo, CP 30370 Chiapas, Mexico; 3 Department of Global Health and Population, Harvard School of Public Health, 677 Huntington Ave, Boston, MA 02115 United States of America; 4 College of Osteopathic Medicine, Touro University California, 1310 Club Drive, Mare Island, Vallejo, CA 94592 United States of America; 5 Department of Obstetrics and Gynecology, Oregon Health and Science University, Portland, OR United States of America; 6 Institute of Public Health, University of Heidelberg, Heidelberg, Germany

**Keywords:** Health promotion, Preventive health services, Human resources planning, Health manpower, Health workforce, Health personnel, Primary health care, Mexico

## Abstract

**Background:**

The purpose of this study was to estimate the gap between the available and the ideal supply of human resources (physicians, nurses, and health promoters) to deliver the guaranteed package of prevention and health promotion services at urban and rural primary care facilities in Mexico.

**Methods:**

We conducted a cross-sectional observational study using a convenience sample. We selected 20 primary health facilities in urban and rural areas in 10 states of Mexico. We calculated the available and the ideal supply of human resources in these facilities using estimates of time available, used, and required to deliver health prevention and promotion services. We performed descriptive statistics and bivariate hypothesis testing using Wilcoxon and Friedman tests. Finally, we conducted a sensitivity analysis to test whether the non-normal distribution of our time variables biased estimation of available and ideal supply of human resources.

**Results:**

The comparison between available and ideal supply for urban and rural primary health care facilities reveals a low supply of physicians. On average, primary health care facilities are lacking five physicians when they were estimated with time used and nine if they were estimated with time required (*P* < 0.05). No difference was observed between available and ideal supply of nurses in either urban or rural primary health care facilities. There is a shortage of health promoters in urban primary health facilities (*P* < 0.05).

**Conclusion:**

The available supply of physicians and health promoters is lower than the ideal supply to deliver the guaranteed package of prevention and health promotion services. Policies must address the level and distribution of human resources in primary health facilities.

## Background

The availability of sufficient human resources (HR) for delivery of health services is a major global policy concern [1]. The health workforce is the social and technical foundation of any health system [2], and the absence or poor distribution [3] of HR can negatively impact both the delivery of health services and the accomplishment of local or national population health goals [4]. The strengthening of primary health care and health promotion services through sufficient human resources has been identified as a high priority area for health systems in many countries [5]. According to the World Health Organization’s (WHO) recent reports, most developing countries, despite important advancements, are still struggling to find clear guidelines for integration of HR into health systems; however, these countries have been experiencing important transformations in recent decades [6, 7].

The Mexican health system is segmented into social security (employment-based), public (Ministry of Health), and private sectors. The health system has evolved through three generations of reforms, which have motivated various actions and strategies for the strengthening of HR. The first reform, in 1943 [2, 8], highlighted the creation of the social security sub-system and focused primarily on making HR available in hospitals.

The second reform, in the 1980s and 1990s, focused on hiring HR for extending coverage through the decentralization of the public sub-system (Ministry of Health) and strengthening of primary health care (PHC) [4, 8, 9]. At that time, “Oportunidades” (formerly PROGRESA, now PROSPERA), a conditional cash transfer program that began in 1997 [10], was specifically designed to encourage demand for health services at the primary care level. Physicians, nurses, and health promoters were hired by the program to provide services to beneficiaries.

The third reform, in the early 2000s, created *Seguro Popular de Salud* (SPS) (2003) to allocate new resources to strengthen primary care and health promotion services in the public sub-system. SPS also aimed to transform the existing curative, hospital-centered health care model to a new one more focused on primary care, prevention, and health promotion. This reform meant to hire and allocate personnel for general hospitals and primary health care facilities in rural and urban areas. SPS slowly rolled out across the country covering by 2012 around 57 million Mexicans [11]. It provides a package of 284 primary and secondary care interventions (CAUSES in Spanish) aimed at improving population health, reducing out-of-pocket expenditures, and satisfying client expectations. SPS serves the population outside the formal sector of the economy who are not eligible for social security (employment-based coverage). In each state, the Health Social Protection Regime (REPSS in Spanish) was created to take responsibility for pooling together the different sources of financing and allocate them according to SPS managerial guidelines to guarantee the provision of services to beneficiary populations.

Seguro Popular greatly expanded access to health services to the uninsured population [12–15], with the gradual introduction of the CAUSES package, which includes both health promotion and preventive services (PPPS) [14, 16]. CAUSES should operate in all Ministry of Health (public) primary health care facilities. The PPPS has 99 guaranteed activities and was structured to cover the needs of different age groups (Table 1). The massive expansion in demand and access to services following implementation of SPS required additional HR for health in all states in the country. However, the newly contracted health personnel were concentrated in urban areas, due to the lack of an explicit and specific HR distribution policy [14, 17], leaving rural facilities with little HR capacity. Thus, the number of health professionals in primary care facilities has not grown significantly, while at the same time an increasingly larger population is demanding health care.

**Table 1:** Guaranteed prevention and health promotion package by age group

Newborns	Children under 9 years	Teens 10–19 years	Women and men 20–59 years	Women and men over 60 years	Pre- and postpartum
1. Risk signs 2. Birth conditions 3. Complete physical examination 4. Birth defects 5. Umbilical cord examination 6. Neonatal screening 7. Eye prophylaxis and vitamin K 8. Vaccination 9. Promotion of breastfeeding and early stimulation 10. Card to follow child	1. Weight and height 2. Complete physical examination 3. Visual acuity 4. Growth and learning disorders 5. Postural problems 6. Family factors of poor prognosis (<5 years) 7. Complete vaccination schedule 8. Micronutrients administration (<5 years) 9. Oral health 10. Healthy nutrition 11. Early stimulation (<5 years) 12. Physical activity and accident prevention 13. Individual and family hygiene education	1. Tuberculosis risk 2. Nutritional status 3. Attention disorders and addictions 4. Complete physical examination 5. Identification of pregnancy 6. Detection of sexually transmitted infections and HIV/AIDS 7. Complete vaccination 8. Reproductive health and contraception 9. Promoting physical activity, oral health, and accident prevention	1. Tuberculosis and BK 2. Sexually transmitted diseases and HIV/AIDS 3. Diabetes, hypertension, overweight, obesity, and osteoporosis 4. Climacteric and menopause (women >40 years) 5. Prostatic disease (men) 6. Complete vaccination 7. Papanicolaou and breast exam (women) 8. Sexual and reproductive health 9. Education for preventing cervical and breast cancer 10. Physical activity, accident prevention, alcohol, and tobacco smoke risk 11. Oral health	1. Visual and hearing problems 2. Prostatic disease 3. Diabetes, hypertension, overweight, obesity, and osteoporosis 4. Tuberculosis risk and BK 5. Signs of cognitive impairment and depression 6. Complete vaccination 7. Breast examination 8. Oral health 9. Physical activity and accident prevention (falls) 10. Information about cervical and breast cancer. 11. Education on acute respiratory infections and vaccines	1. Weight and blood pressure 2. Pregnancy confirmation 3. Pregnancy risks factors 4. Perinatal card 5. Laboratory studies (blood test, syphilis, complete urinalysis, and others) 6. Complete vaccination to pregnant women 7. Micronutrient administration 8. Oral health 9. Parental education 10. Care of the newborn 11. Family planning 12. Health education and treatment to HIV/AIDS

Source: Adapted from Lifeline strategy for Prevention and Health Promotion

In order to transform administrative and financial reforms into concrete actions to improve population health status [15, 18] and health service delivery goals, it is critical to have sufficient human resources and to distribute them appropriately [19]. Evidence on the sufficiency of human resources to meet health goals and objectives is scarce. Information on the link between human resources supply and prevention and promotion services is even more sparse. The purpose of this study was to estimate the gap between the available and the ideal supply of human resources to deliver health prevention and promotion services to the Mexican population served by the public sub-system. We hypothesized that the available supply of human resources is insufficient to ensure the delivery of PPPS in rural and urban primary health care facilities in Mexico.

## Methods

We conducted a cross-sectional observational study and used a convenience sample considering the following criteria: (a) geographic diversity (North, Center, and South), (b) REPSS juridical status (decentralized, deconcentrated, or integrated), and (c) level of REPSS performance (percentage of the population affiliated to SPS and percentage of population with SPS that use health services). Ten states were included (Morelos, Ciüdad de México, Hidalgo, Querétaro, Guerrero, Baja California, Jalisco, Campeche, Zacatecas, and Estado de México). In each state, we selected two primary care facilities (PHFs) that provided a prevention and health promotion package since initiation of SPS in 2003 (Lifeline Program). One rural and one urban PHF was included (we used the classification of urban/rural PHF established by the General Directorate of Health Information). However, Mexico City is completely urban and Jalisco did not have rural primary health facilities that had implemented the package by 2003, so in these states, we included only urban facilities.

**Fig. 1:**
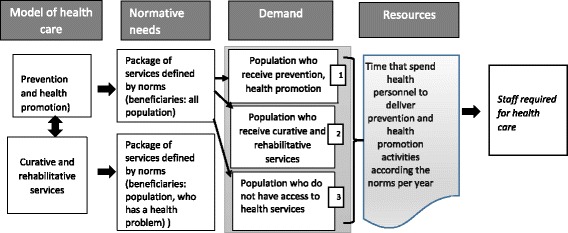
Sub-model for calculating health-staffing requirements from normative needs. This study only considers population who received service in PHF (1y2). Source: adapted from sub-personal requirement model proposed by division of family

Our analysis is guided by the service delivery target model, which is based on demand of health services [20–23] and the availability of human resources to deliver health services. To estimate the demand of preventive and health promotion services, we employed the normative-need approach [24], which relies on an expert opinion about which health services an individual should receive over 1 year (Fig. 1). We focused on all 99 prevention and promotion activities that are included in the guaranteed package for all age groups [16] included in CAUSES and implemented in all primary care facilities (Table 1).

Our operational variables to estimate HR were available and ideal supply, both estimated from time variables. Available supply (As) was estimated as the average of payable number of hours stipulated in staff labor contracts (physicians, nurses, and health promoters) in primary health facilities in 2009. Ideal supply (Is) was estimated from time used and required to perform health promotion and prevention activities (Table 1). Time used is the time that staff use to deliver health prevention and promotion activities while time required is the time that staff considered suitable to deliver prevention and health promotion activities. To estimate our operational variables, we followed several steps (Table 2):

**Table 2:** Median of time used and required by individual per year according to age group to deliver the guaranteed package of prevention and health promotion. Mexico, 2009

Age group	Number of prevention and health promotion activities	Total of population attended in PHFs	Median of time used by individual per year (min)				Median of time required by individual per year (min)				Differences			
			Total (a1)	Physician (a2)	Nurse (a3)	HP (a4)	Total (b1)	Physician (b2)	Nurse (b3)	HP (b4)	a1 − b1	a2 − b2	a3 − b3	a4 − b4
Newborns	10	5 601	55.50	24.00	27.00	5.00	90.00	41.00	43.50	10.00	−34.50	−17.00	−16.50	−5.00
Children under 5 years	10	41 094	61.50	13.50	33.50	15.50	94.00	20.00	46.00	22.50	−32.50	−6.50	−12.50	−7.00
Children from 5 to 9 years	10	24 266	71.00	30.00	20.00	14.00	94.50	40.00	30.00	22.50	−23.50	−10.00	−10.00	−8.50
Teen from 10 to 19 years	10	50 508	97.50	60.00	17.50	27.50	131.00	77.50	25.00	35.00	−33.50	−17.50	−7.50	−7.50
Women from 20 to 59 years	11	76 839	97.50	40.00	25.00	35.50	165.40	57.50	37.50	76.50	−67.90	−17.50	−12.50	−41.00
Men from 20 to 59 years	11	39 459	83.50	37.50	32.50	25.00	152.50	51.50	30.00	62.50	−69.00	−14.00	2.50	−37.50
Women and men over 60 years	10	16 591	100.00	52.50	39.50	15.00	162.50	70.00	72.50	20.00	−62.50	−17.50	−33.00	−5.00
Pregnant women														
First visit	10	7 610	61.50	46.00	10.00	5.00	85.50	67.00	10.00	9.00	−24.00	−21.00	0.00	−4.00
Subsequent visit	7	5 076	46.50	36.50	10.00	0.00	58.50	47.00	10.00	0.00	−12.00	−10.50	0.00	0.00
Postnatal care	10	1 483	58.50	27.50	35.00	0.00	91.50	37.00	50.00	0.00	−33.00	−9.50	−15.00	0.00
All groups	99	268 527	73.00	40.00	25.00	22.00	105.00	51.00	35.00	30.00	−32.00	−11.00	−10.00	−8.00

First, we organized a group of experts all of whom were knowledgeable about prevention and health promotion, having at least 10 years of experience working in primary care facilities and implementing prevention and promotion activities included in the Lifeline Program [16]. The group included two physicians, two nurses, three health promoters, two primary health technicians, two local health coordinators, and one health promotion coordinator at the state level. Experts checked all 99 activities and assigned each one to a single occupational category (physician, nurse, or primary care technician).

Second, available supply was estimated from the number of personnel available (physicians, nurses, and health promoters) at each primary care facility in 2009 (using Ministry of Health official information) [25] multiplied by 200 working days per year and six working hours per day. These figures were added up to obtain the total of annual hours available as a variable of available supply.

Third, we developed an instrument to estimate the time used and the time required. Health workers reported the time used and the time required to perform a single activity from the package of prevention and health promotion. The instrument was piloted in two PHFs in the State of México not included in the study. During the pilot phase, the researchers measured the time used to perform a group of activities (16 of the 99), and then we compared this time with the time reported by health staff. Eighty percent of the time measured to perform activities were very similar to the time reported, and the remaining 20% were not statistically significantly different.

Next, we estimated the ideal supply. Teams working in each of the participating primary care facilities estimated by consensus the amount of time used and time required for performing each of the 99 activities. The age and sex of the individuals were very important, because the package of prevention and health promotion has different annual frequencies and different number of activities whether the subject is a child, adolescent, adult, elderly, man, or woman. Estimation of time used and required by activity according to age and sex was multiplied by the total number of individuals who received care in PHFs in 2009. Then, we added up these figures to obtain the total of annual hours from time used and time required to obtain two variables of ideal supply, one estimated from time used and another from time required. The available and ideal supply estimations were made under the assumption that all personnel at each facility spent all their working time to perform health promotion and prevention activities, as we had no reliable estimates of the distribution of working time to perform health promotion and prevention services.

Fourth, we calculated available and ideal supply by age groups, personnel type, and rural/urban areas and information is presented using descriptive statistics. To compare similarities and differences, we used median differences (Table 2) as the distribution of data was not normal. The null hypothesis was that the median of available supply is equal to the median of ideal supply (time used and required). We used the Wilcoxon matched-pairs signed-ranks test to compare available supply with ideal supply (available supply with ideal supply estimated from time used or available supply with ideal supply estimated from time required). Friedman test was used to compare available and ideal supply estimated from used time and ideal supply estimated from time required. Our samples are non-independent (time estimates come from the same teams); these tests account for dependence of the estimates [26]. After, we estimated available and ideal supply into number of human resources (annual hours estimated were divided by 1200 working hours per health worker per year).

Finally, we conducted a sensitivity analysis to test whether the non-normal distribution of our time data biased our estimates of available and ideal supply of human resources. We used the gamma density function (Table 3) because of the non-normal distribution of the variables and tested the following assumptions: (a) asymmetrical distribution, (b) positive asymmetry, (c) no values lower than zero, and (d) the existence of a high degree of variability. We estimated alpha, the peak of the frequency distribution. A smaller alpha value indicates more highly skewed data, with variation so great as to render the median not useful as a method to summarize the distribution. No data were found to have an alpha <1.33, which indicates that our use of the gamma distribution fit our data and the median is a valid way to summarize our data.

**Table 3:** Estimation of variables

e) Density function $\text{\textit{\textsf{f}}}(\text{\textit{\textsf{x}}})=\frac{1}{\beta^{\alpha}\overline{|(\alpha)}}\ \text{\textit{\textsf{x}}}^{\alpha-1}\text{\textit{\textsf{e}}}^{\text{\textit{\textsf{x}}}/\beta}$ where *x* > 0; *α* and *β* are positive parameters *x:* used and required time function measured in minutes *α*: form parameter *β*: scale parameter *f(x)*: gamma function *e*: exponential function	h) Sensitivity index: $S=\frac{1}{1-\alpha }$ *tu =* used time *tr =* required time

## Results

Of the 20 PHFs, 60% (12) were located in an urban area and 40% (8) in a rural one. A total of 866 health workers were working on PHFs, 57% (CI 53–60; *n* = 489) delivered health services and 43% (CI 40–47; *n* = 377) worked as administrative staff. Of the workers who delivered health services, 45% (CI 41–50; *n* = 220) were nurses; 36% (32–40; *n* = 176) physicians; 8% (CI 06–11; *n* = 39) health promoters; 3% (CI 2–5; *n* = 15) nutritionists, psychologists, or social workers; and 8% (CI 6–11; *n* = 39) dentists or medical students in social service. The total population that received care in PHFs in 2009 accounted for 268 527 individuals, 87% (*n* = 233 618) received care in urban and 13% (*n* = 34 909) in rural PHFs.

The median of time used to deliver all activities from the guaranteed package of prevention and health promotion per year by individuals across age and gender groups was lower than the median of time required; a difference of −32 min was observed between them (Table 2). In the case of physicians, the median difference between time used and required was −11 min; for nurses, −10 min; and for primary health technicians (PHTs), −8 min. These results show that the time used is lower than the time required to perform prevention and health promotion activities across all age groups and occupational categories.

By age, the largest gaps between time used and time required were observed for women and men from 20 to 59 and women and men over 60 years; the difference was more than 1 h. For newborns, children from 1 to 4 years, teens from 10 to 19 years, and postpartum women, the difference was almost 30 min. Differences lower than 24 min were observed for children of 5–9 years and subsequent queries of pregnant women (Table 2).

The analysis of available and ideal supply by occupational categories using the Friedman chi-square test suggests the distributions (Table 4) of these variables for physicians are statistically different (*P* = 0.001). The Wilcoxon test that compared available supply with ideal supply estimated by time used (*P =* 0.003) and time required (*P =* 0.002) is statistically significant. Therefore, the negative outcome difference confirms that available supply is lower than ideal supply.

**Table 4:** Available and ideal supply to deliver the guaranteed package of prevention and health promotion at PHFs. Mexico, 2009

Variables	Physician			Nurses			Health promoters		
	Available supply (As)	Ideal supply (Is)		Available supply (As)	Ideal supply (Is)		Available supply (As)	Ideal supply (Is)	
	Annual hours available 2009	Annual hours estimated time used	Annual hours estimated time required	Annual hours available 2009	Annual hours estimated timeused	Annual hours estimated time required	Annual hours available 2009	Annual hours estimated time used	Annual hours estimated time required
	(a)	(a1)	(a2)	(b)	(b1)	(b2)	(c)	(cl)	(c2)
Urban PHFs (*n =* 12)									
Total hours	141 600	373 462.6	562 069.7	220 800	231 150.6	332 150.6	27 600	243 449.9	406 915.0
Median (h)	12 000	22 624.2	35 876.9	17 400	12 654.8	21 913.9	0	16 749.0	32 658.9
As − Is		−10 624.2	−23 876.9		4 475.3	4 513.9		−16 749.0	−32 658.9
Wilcoxon (*P* value)		0.0029	0.002 2		0.209 4*	0.099 5*		0.002 2	0.002 2
Friedman (*P* value)			0.001 3			0.001 2			0.028 0
Rural PHFs (*n =* 8)									
Total hours	30 000	628 853.6	84 632.2	38 400	40 127.2	57 173.1	1 200	35 627.8	52 308.2
Median	1 800	4 079.7	5 460.1	1 200	2 173.6	3 182.4	0	2 151.3	3 714.2
As − Is		−2 279.7	−3 660.1		−973.6	−1 982.4		−2 151.3	−3 714.2
Wilcoxon (*P* value)		0.049 9	0.017 3		NA	NA		NA	NA
Friedman (*P* value)			0.026 2			0.083 7*			0.170 5*
Total PHFs (*n =* 20)									
Total hours	171 600	436 316.2	646 701.9	259 200	271 545.6	389 393.7	28 800	279 077.7	459 223.2
Median	6 000	12 478.5	16 510.6	10 800	7 904.6	11 321.3	0	7 220.7	9 252
As − Is		−6 478.5	−10 510.6		2 895.4	−521.3		−7 220.7	−9 252
Wilcoxon (*P* value)		0.000 4	0.000 1		0.262 7*	0.018 7		0.000 1	0.000 1
Friedman (*P* value)			0.000			0.000 1			0.016 7

* = <0.05

The supply analysis for nurses (Table 4) suggests that available and ideal supply are different (Friedman *P =* 0.0001), but the Wilcoxon test shows that available and ideal supply estimated by time used (*P =* 0.252) are not different. Available supply is lower than ideal supply estimated by time required (*P =* 0.019). No differences were found in nurse supply when analyzed by urban and rural PHFs (*P >* 0.05).

The supply analysis for health promoters (Table 4) suggests that available supply and ideal supply are different (Friedman *P =* 0.0167). The Wilcoxon test that compares available with ideal supply estimated by time used (*P =* 0.0001) and ideal supply estimated by time required (*P =* 0.0001) shows differences between them. Also, available with ideal supply estimates by time used and required (*P =* 0.0022) are different in urban PHFs. Therefore, available supply is lower than ideal supply and no differences on HP supply were found in rural PHFs (*P >* 0.05).

For health workers, the median of available supply across PHFs was 5 physicians, the median of ideal supply estimated by time used was 10.4, and the median estimated by time required was 13.76 physicians (Table 5). Results show that the ideal supply of physicians is greater than the available supply for urban and rural PHFs. However, the gap of physicians is greater for urban PHFs. In the case of the nurses, we did not find any differences between available and ideal supply.

**Table 5:** Available and ideal supply expressed on number of doctors and primary health promoters. Mexico, 2009

Primary health facilities	Number of doctors			Number of health promoters		
	Available supply	Ideal supply		Available supply	Ideal supply	
		Time used	Time required		Time used	Time required
Urban PHFs						
Tapalpa	6.0	5.5	8.7	0.0	4.0	6.2
Jesus Rosal	11.0	60.6	108.9	0.0	37.3	65.7
Satellite	2.0	3.2	4.7	0.0	1.3	2.4
Coapa	10.0	11.1	16.1	0.0	6.6	8.3
GR. Millan	10.0	15.3	29.5	0.0	6.8	13.3
Zacatecas	10.0	24.8	30.3	0.0	14.0	27.1
W. Escalante	10.0	24.3	36.0	0.0	17.5	27.3
Industrial	13.0	43.2	48.4	0.0	25.3	35.0
Rena II	3.0	4.8	6.1	0.0	3.1	6.5
Pedro Escobedo	23.0	81.0	119.9	1.0	55.2	79.8
Toluca	16.0	22.4	33.4	9.0	17.8	36.7
San Rafael	4.0	13.0	26.3	13.0	13.9	30.9
Median	10.0	18.9	29.9	0.0	14.0	27.2
Rural PHFs						
Juanacatlan	2.0	9.7	11.4			
Mineral Chico	3.0	6.8	10.5			
Cuentepec	4.0	2.0	2.9			
Sta Elena	1.0	2.1	2.2			
Koben	1.0	1.4	2.6			
G. Victoria	12.0	23.6	31.8			
R02 Kilometro 30	1.0	4.3	5.2			
P. Coyote	1.0	2.5	3.9			
Median	1.5	3.4	4.6			
General median PHF	5.0	10.4 −5.4	13.8 −8.8			

In the case of health promoters, the median of the available supply was “zero”; only in two PHFs were these health workers found. The ideal supply in urban PHFs is 9.96 HP when it was estimated by time used and 27.22 HP when it was estimated by time required. We did not estimate health promoters for rural PHFs because the available supply is the same with the ideal supply (Table 4).

## Discussion

Our results show that available supply is lower than ideal supply of HR meaning that the amount of HR available is not enough [27] to deliver preventive and health promotion services. However, the real scenario is likely worse, because this study assumed that health personnel are dedicated exclusively to performing activities to deliver the package of prevention and health promotion services, but in reality, they use only part of their time for this service provision. The gap of physicians in urban and rural PHFs [28] is clear but it is important to highlight that it is far greater in urban areas than in rural ones. The larger gap observed in urban areas compared to rural areas can be explained by a higher demand of services due to population density, the fact that services are available more hours a day (12 in urban areas compared to 6 in rural areas) and over weekends [29], implying a greater demand for HR [30]. This highlights the importance of considering demand for services as well as the volume of the catchment population as a criterion when distributing HR for health.

Internationally, the lack of human resources in primary care facilities has been widely documented [31–34], especially on medical staff and rural areas [35, 36]. The absence of prioritization of policies for prevention and health promotion is one possible explanation for this maldistribution of human resources [37]. Despite the transformation of health service demand, dominated today by chronic diseases [38, 39], in developed and developing countries, health promotion has not yet been properly included in national and local agendas.

Furthermore, international literature has explained the low availability of physicians in rural areas because of attractive job opportunities outside their home country, the lack of professional development [40, 41], the inequalities in the distribution of health workers [42], and a persistent lack of policy to prioritize the distribution of HR to rural areas [43]. On the other hand, physicians have historically expressed low interest to work in rural areas. For example, in Ayacucho, Peru, physicians are five times more likely to choose an urban area than a rural one [44]. In the United States of America, poor recruitment is likely to be the principal reason for short length of stay in rural areas [45], and in Canada, low salary is the main determinant [46].

Based on this trend, international agencies have called for the strengthening of the primary health care model [40, 47]. This model should be centered in health promotion and preventive services [39–48] and needs not only more [42–49] but also well-trained health personnel and the right skill mix [50] to deliver preventive and health promotion services. Therefore, governments should develop strategies and policies for health personnel retention in PHFs [46, 51, 52], which is one of the biggest challenges of health systems.

In the area where this research was conducted, PHF health promoters were not available. This absence can be explained because hiring of these personnel has remained stagnant in recent years. Those who are retired are being replaced by administrative staff. This situation intensifies the lack of this kind of personnel to deliver prevention and health promotion services in PHFs.

One of the limitations of this study is self-reported time team consensus measurements. We were unable to accurately calculate time to perform prevention and promotion activities and thus chose to assume that 100% of time of health workers was dedicated to such activities. This means that our results are likely biased towards the null (no) difference between available and ideal supply since personnel also devote time to curative and administrative activities on PHFs. The Ministry of Health of Mexico should consider to revise the structure of its databases to provide more accurate data that maybe used for research and policy-making purposes.

## Conclusions

Based on a conservative analysis, we used data from teams of health care providers and conservative estimates to identify a gap in the current/available and ideal supply of physicians in urban and rural areas, and health promoters in urban areas to deliver a package of prevention and health promotion services. To improve service delivery, several things are needed: (1) an increase of the HR at the PHFs, (2) ensuring complete staff at the PHFs (physicians, nurses, and health promoters), (3) improving their set of skills about prevention and health promotion, and (4) developing policies to retain personnel at PHFs.

## Abbreviations

As: Available supply
CAUSES: Package of care interventions
HR: Human resources
Is: Ideal supply
PHC: Primary health care
PHFs: Primary health facilities
PHTs: Primary health technicians
REPSS: Health Social Protection Regime
SPS: Public health insurance (*Seguro Popular de Salud*)
WHO: World Health Organization

## References

[1] Kabene SM, Orchard C, Howard JM, Soriano MA, Leduc R (2006). The importance of human resources management in health care: a global context. Hum Resour Health.

[2] Frenk J, Sepúlveda J, Gómez-Dantés O, Knaul F (2003). Evidence-based health policy: three generations of reform in Mexico. Lancet.

[3] López Pardo y Cándido M. La reforma sanitaria en América Latina y el Caribe. Rev Cubana Salud Pública. 1997; 23: 17-31.

[4] Frenk J, Robledo-Vera C, Nigenda-López G, Ramírez-Cuadra C, Galván-Martínez O, Ramírez-Avila J (1990). Políticas de formación y empleo de médicos en México: 1917-1988. Salud Publica Mex.

[5] Regan S, Wong ST, Watson DE (2010). Public perspectives on health human resources in primary healthcare: context, choices and change. Healthcare Policy.

[6] World Health Organization. Colaboremos por la salud: Informe sobre la salud del mundo 2006. http://www.who.int/whr/2006/whr06_es.pdf. Accessed 3 Mar 2015.

[7] Giraldo Osorio A, Vélez Álvarez C (2012). Primary health care: challenges for implementation in Latin America. Aten Primaria.

[8] Soberon G (2001). La reforma de salud en México. Gac Med Mex.

[9] Gómez-Dantés O, Gómez-Jáuregui J, Inclán C (2004). La equidad y la imparcialidad en la reforma del sistema mexicano de salud. Salud Pública Mex.

[10] Coordinación Nacional de Prospera Programa de Inclusión Social. Available on: https://www.prospera.gob.mx/swb/es/PROSPERA2015/Quees_PROSPERA. Accessed 7 Apr 2015.

[11] INEGI. Derechohabiencia y uso de servicios de salud. Población protegida por los servicios de salud, 2000 a 2015. http://www3.inegi.org.mx/sistemas/sisept/default.aspx?t=msoc01&s=est&c=22594. Accessed 3 Oct 2016.

[12] Gómez Dantés O, Ortiz M (2004). Seguro Popular de Salud: siete perspectivas. Salud Publica Mex.

[13] Fineberg H (2007). Reforma de salud en México: un trabajo que avanza. Salud Publica Mex.

[14] Frenk J (2007). Tender puentes: lecciones globales desde México sobre políticas de salud basadas en evidencias. Salud Publica Mex.

[15] Frenk J, González-Pier E, Gómez-Dantés O, Lezana MA, Knaul FM (2007). Reforma integral para mejorar el desempeño del sistema de salud en México. Salud Publica Mex.

[16] Secretaría de Salud. Prevención y Promoción de la salud durante la línea de vida. México 2003. http://www.insp.mx/Portal/Centros/ciss/nls/caravanas/material_didactico/mod2_8.pdf. Accessed 15 Mar 2011.

[17] Nigenda G (2013). Servicio social en medicina en México. Una reforma urgente y posible. Salud Publica Mex.

[18] Victora C, Barreto M, do Carmo Leal M, Monteiro C, Schmidt M, Barros F, et al. Health conditions and health-policy innovations in Brazil: the way forward. Lancet (London, England) [serial on the Internet]. 2011;377(9782):2042–53. [cited July 25, 2017]. Available from: MEDLINE with Full Text. 10.1016/S0140-6736(11)60055-X 21561659

[19] Doubova SV, Ramírez-Sánchez C, Figueroa-Lara A, Pérez-Cuevas R (2013). Recursos humanos para la atención de pacientes con diabetes en unidades de medicina familiar del Instituto Mexicano del Seguro Social. Salud Publica Mex.

[20] Dreesch N, Dolea C, Dal Poz MR (2005). An approach to estimating human resource requirements to achieve the Millennium Development Goals. Health Policy Plan.

[21] Chorny A, Novaro S, Stulhman L, Bernachi M (1976). Método para el análisis y la estimación de recursos humanos para los programas de atención materno infantil. Educ Med Salud.

[22] Bärnighausen T, Bloom D. Changing research perspectives on the global health workforce. National Bureau of Economic Research. Massachusetts 2009. Working Paper 15168 http://www.nber.org/papers/w15168. Accessed 14 Sept 2015.

[23] Barranquero A y González A. Una revisión de modelos econométricos aplicados al análisis de demanda y utilización de servicios sanitarios. Hacienda Pública Española. 2005;(173):129–62.

[24] Comisión Nacional de Protección Social en Salud. Catálogo Universal de Servicios de Salud 2010. http://seguropopular.col.gob.mx/segpop/pdf/causes2010.pdf. Accessed 4 Aug 2010.

[25] Secretaria de Salud. Condiciones Generales de trabajo. http://www.inmegen.gob.mx/tema/cms_page_media/589/CONDICIONES%20GENERALES%20DE%20TRABAJO%202011-2013.pdf. Accessed 4 Oct 2012.

[26] University of California, Los Angeles. What statistical analysis should I use? Statistical analyses using Stata. http://www.ats.ucla.edu/stat/stata/whatstat/whatstat.htm. Accessed 2 Mar 2016.

[27] Chen PG, Mehrotra A, Auerbach DI (2014). Do we really need more physicians? Responses to predicted primary care physician shortages. Med Care.

[28] Kaiyuan Z, Xinyi Z, Yi D, Duolao W, Zhou L, Yu M, et al. Inequality trends of health workforce in different stages of medical system reform (1985-2011) in China. Human Resources For Health [serial on the Internet]. 2015;131–8. [cited July 25, 2017]. Available from: Academic Search Premier. 10.1186/s12960-015-0089-0 PMC4673776 26645960

[29] Ensor T, Cooper S (2004). Overcoming barriers to health service access: influencing the demand side. Health Policy Plan.

[30] Whitford D, Smith T, Newbury J (2012). The South Australian Allied Health Workforce survey: helping to fill the evidence gap in primary health workforce planning. Aust J Prim Health.

[31] Thoresen SH, Fielding A (2010). Inequitable distribution of human resources for health: perceptions among Thai healthcare professionals. Qual Prim Care.

[32] Pallikadavath S, Singh A, Ogollah R, Dean T, Stones W (2013). Human resource inequalities at the base of India’s public health care system. Health Place.

[33] dos Reis Moreira ÉC, O’Dwyer G (2013). An analysis of actions to promote health in underprivileged urban areas: a case in Brazil. BMC Fam Pract.

[34] Phalkey R, Dash SR, Mukhopadhyay A, Runge-Ranzinger S, Marx M. Prepared to react? Assessing the functional capacity of the primary health care system in rural Orissa, India to respond to the devastating flood of September 2008. Glob Health Action. 2012;5(1):10964. 10.3402/gha.v5i0.10964 PMC3307669 22435044

[35] McQuide PA, Kolehmainen-Aitken RL, Forster N (2013). Applying the workload indicators of staffing need (WISN) method in Namibia: challenges and implications for human resources for health policy. Hum Resour Health.

[36] Daviaud E, Chopra M (2008). How much is not enough? Human resources requirements for primary health care: a case study from South Africa. Bull World Health Organ.

[37] Nyamhanga T, Frumence G, Mwangu M, Hurtig AK. ‘We do not do any activity until there is an outbreak’: barriers to disease prevention and health promotion at the community level in Kongwa District, Tanzania. Global health action. 2014;7(1):23878. 10.3402/gha.v7.23878 PMC4119291 25084832

[38] Córdova-Villalobos JÁ, Barriguete-Meléndez JA, Lara-Esqueda A, Barquera S, Rosas-Peralta M, Hernández-Ávila M, Aguilar-Salinas CA (2008). Las enfermedades crónicas no transmisibles en México: sinopsis epidemiológica y prevención integral. Salud Publica Mex.

[39] Bodenheimer T, Chen E, Bennett HD (2009). Confronting the growing burden of chronic disease: can the US health care workforce do the job?. Health Aff.

[40] Alameddine M, Saleh S, El-Jardali F, Dimassi H, Mourad Y (2012). The retention of health human resources in primary healthcare centers in Lebanon: a national survey. BMC Health Serv Res.

[41] Garson JR (2013). New systems of care can leverage the health care workforce: how many doctors do we really need?. Acad Med.

[42] Gravelle H, Sutton M (2013). Inequality in the geographical distribution of GPs in England and Wales 1974-1995. J Health Serv Res Policy.

[43] Gupta N, Zurn P, Diallo K, Dal Poz MR (2003). Uses of population census data for monitoring geographical imbalance in the health workforce: snapshots from three developing countries. Int J Equity Health.

[44] Miranda J, Diez-Canceco F, Lema C, Lescano A, Legarde M, Blaauw D, Hicho L (2012). Stated preference of doctors for choosing a job in rural areas of Peru: a discrete choice experiment. PloS One.

[45] Pathman DE, Konrad TR, Dann R, Koch G (2004). Retention of primary care physicians in rural health professional shortage areas. Am J Public Health.

[46] Wranik DW, Durier-Copp M (2010). Physician remuneration methods for family physicians in Canada: expected outcomes and lessons learned. Health Care Anal.

[47] Organización Panamericana de la Salud.Atención primaria de salud y desarrollo de recursos humanos. Unidad de desarrollo de recursos humanos. 2003. Accessed 20 Oct 2013.

[48] Starfield B, Shi L, Macinko J (2005). Contribution of primary care to health systems and health. Milbank Q.

[49] Schwartz MD (2012). Health care reform and the primary care workforce bottleneck. J Gen Intern Med.

[50] Rey-Gamero AC, Acosta-Ramírez N (2013). El enfoque de competencias para los equipos de Atención Primaria en Salud. Una revisión de literatura. Rev Gerencia Pol Salud.

[51] Shroff ZC, Murthy S, Rao KD (2013). Attracting doctors to rural areas: a case study of the post-graduate seat reservation scheme in Andhra Pradesh. Indian J Community Med.

[52] Russel DJ, McGrail MR, Humphreys JS, Wakerman J (2012). What factors contribute most to the retention of general practitioners in rural and remote areas?. Aust J Prim Health.

